# Adaptive Filtering for Channel Estimation in RIS-Assisted mmWave Systems

**DOI:** 10.3390/s25020297

**Published:** 2025-01-07

**Authors:** Shuying Shao, Tiejun Lv, Pingmu Huang

**Affiliations:** 1 School of Information and Communication Engineering, Beijing University of Posts and Telecommunications (BUPT), Beijing 100876, China; shaosy@bupt.edu.cn; 2 School of Artificial Intelligence, Beijing University of Posts and Telecommunications (BUPT), Beijing 100876, China

**Keywords:** channelestimation (CE), reconfigurable intelligent surfaces (RISs), adaptive filtering, sparse mmWave systems

## Abstract

The advent of millimeter-wave (mmWave) massive multiple-input multiple-output (MIMO) systems, coupled with reconfigurable intelligent surfaces (RISs), presents a significant opportunity for advancing wireless communication technologies. This integration enhances data transmission rates and broadens coverage areas, but challenges in channel estimation (CE) remain due to the limitations of the signal processing capabilities of RIS. To address this, we propose an adaptive channel estimation framework comprising two algorithms: log-sum normalized least mean squares (Log-Sum NLMS) and hybrid normalized least mean squares-normalized least mean fourth (Hybrid NLMS-NLMF). These algorithms leverage the sparse nature of mmWave channels to improve estimation accuracy. The Log-Sum NLMS algorithm incorporates a log-sum penalty in its cost function for faster convergence, while the Hybrid NLMS-NLMF employs a mixed error function for better performance across varying signal-to-noise ratio (SNR) conditions. Our analysis also reveals that both algorithms have lower computational complexity compared to existing methods. Extensive simulations validate our findings, with results illustrating the performance of the proposed algorithms under different parameters, demonstrating significant improvements in channel estimation accuracy and convergence speed over established methods, including NLMS, sparse exponential forgetting window least mean square (SEFWLMS), and sparse hybrid adaptive filtering algorithms (SHAFA).

## 1. Introduction

The integration of millimeter-wave (mmWave) technology with massive multiple-input multiple-output (MIMO) systems in modern wireless cellular networks offers significant potential to meet future high data rate demands [1]. The mmWave frequency band provides distinct advantages, including enhanced capacity, higher data transmission speeds, and reduced latency. Moreover, the shorter wavelength associated with this frequency band allows for reduced antenna spacing, which subsequently enhances the overall efficiency of communication systems. Conversely, massive MIMO mitigates propagation losses and improves spectral efficiency by exploiting the significant gains provided by large antenna arrays. However, in mmWave systems, the quality of service (QoS) may deteriorate significantly when the line-of-sight (LoS) path is obstructed. To address these challenges, reconfigurable intelligent surface (RIS) technology has been proposed. RIS comprises numerous passive reflective elements that adjust both the phase and amplitude of incoming signals to modify the wireless propagation environment. Additionally, since RIS operates passively, it exhibits exceptionally low power consumption [2]. This functionality allows RIS to reflect signals where the direct LoS link between the transmitters and receivers is obstructed, thereby establishing a virtual LoS path. Potential applications of mmWave massive MIMO combined with RIS include improving coverage at the cell’s edge and facilitating cost-effective, energy-efficient communication solutions. Nonetheless, accurate acquisition of channel state information (CSI) is essential for fully leveraging the advantages of RIS-enhanced mmWave systems. Precise CSI is imperative for optimizing the design of both the precoding matrix and the RIS reflection coefficients, thus enhancing the reliability of data transmission. However, due to the passive characteristics of RIS elements and the substantial number of reflective components involved, the acquisition of CSI remains a complex and resource-intensive task [3].

In contemporary wireless communication systems, there is a growing focus on channel estimation methods to maximize the potential of RIS in enhancing overall system performance. Reference [4] presents a channel estimation framework based on the parallel factor decomposition, employing an iterative estimation algorithm that utilizes alternating least squares and vector approximate message passing, while [5] reformulates the channel estimation problem as an image super-resolution extraction task, utilizing convolutional neural networks for its resolution. However, these methods do not consider the unique characteristics of mmWave systems. In mmWave communications, the channel exhibits significant sparsity due to limited scattering and constrained propagation paths, with most channel coefficients approaching or reaching zero [6]. Specialized adaptive filtering algorithms for sparse channel estimation have been developed to mitigate this sparsity. These algorithms utilize adaptive filters to update the channel, effectively estimating sparse channels iteratively. Furthermore, these algorithms can dynamically adjust based on received data, allowing them to adapt to variations in the communication environment. This adaptability is crucial for RIS-enabled massive MIMO systems, where communication conditions fluctuate across time and location. Consequently, adaptive sparse filtering techniques have garnered considerable interest for their ability to leverage channel sparsity, offering the potential for improved performance in wireless communication systems.

The classical least mean squares (LMS) algorithm in adaptive filtering has received significant attention for its simplicity in implementation and fast convergence rates [7]. However, the standard LMS algorithm is sensitive to the scaling of input signals, making it challenging to determine an appropriate step size that ensures stability. The normalized least mean squares (NLMS) algorithm was developed to address this limitation, which adjusts the input signal power by normalization [8]. Nevertheless, conventional adaptive filtering methods such as LMS and NLMS fail to fully exploit the intrinsic sparsity of mmWave channels, rendering them less effective for sparse channel estimation tasks. Drawing inspiration from compressed sensing (CS) theory [9], sparse channel estimation can be performed by introducing a norm constraint into the cost function of the LMS algorithm. For example, the zero-attraction LMS (ZA-LMS) algorithm proposed in [10] incorporates an $\ell_{1}$ norm constraint to apply uniform zero attraction (ZA) for sparse channel estimation. However, this technique enforces identical zero attraction across all channel coefficients without differentiation. To overcome this limitation, the reweighted ZA-LMS (RZA-LMS) algorithm was introduced in [10], allowing selective zero attraction for particular coefficients. Subsequent research into zero-attraction techniques has developed several sparse LMS algorithms with various norm constraints, including the $\ell_{p}$ norm and approximate $\ell_{0}$ norm constraints [11,12]. Capitalizing on the advantages of traditional NLMS algorithms, zero-attraction techniques have been extended to NLMS, leading to the creation of ZA-NLMS and RZA-NLMS algorithms [13]. However, in low signal-to-noise ratio (SNR) environments, the performance of LMS algorithms often becomes unstable. Methods incorporating higher-order moments of the estimation error have been proposed to mitigate this instability. One prominent technique, the least mean fourth (LMF) algorithm, utilizes the fourth-order moment of the estimation error in its cost function and represents an essential advancement in higher-order adaptive filtering methods. Studies have shown that the LMF algorithm performs robustly under low SNR conditions. To take advantage of the sparse nature of the channels, zero-attraction techniques have also been incorporated into LMF algorithms, resulting in the zero-attraction LMF (ZA-LMF), reweighted ZA-LMF (RZA-LMF), and $\ell_{0}$ norm-constrained LMF ($\ell_{0}$-LMF) algorithms [14,15]. Although these advancements represent significant progress, further refinement is required to optimize these sparse algorithms for fully leveraging the sparse characteristics of mmWave channels, especially in real-world applications.

This study concentrates on RIS-assisted mmWave massive MIMO systems, aiming to leverage the inherent sparsity characteristic of mmWave channels. We propose an adaptive filtering framework designed to tackle the challenges of sparse channel estimation. We introduce two novel algorithms, emphasizing the critical role of adaptive filtering in enhancing channel estimation performance and providing insights that could facilitate advancements in mmWave communication technologies. The primary contributions of this research are summarized as follows:

- We present two advanced adaptive algorithms for sparse channel estimation, Log-Sum NLMS and Hybrid NLMS-NLMF, specifically designed for RIS-assisted mmWave massive MIMO systems. These algorithms effectively address the significant sparsity of mmWave channels and the variability in signal-to-noise ratio (SNR) environments by integrating traditional adaptive filtering techniques with advanced sparse signal processing techniques. As a result, these algorithms enhance the accuracy and adaptability of channel estimation across varying SNR levels while accelerating convergence, thereby significantly improving the overall performance of mmWave communication systems.
- The Log-Sum NLMS algorithm improves upon the conventional NLMS approach by incorporating a log-sum penalty term into its cost function. This modification introduces a zero-attraction mechanism within the iterative estimation process that effectively pulls small channel coefficients toward zero, exploiting channel sparsity. This significantly enhances the performance of the Log-Sum NLMS algorithm in sparse channel estimation, facilitating faster convergence and reducing estimation errors.
- Building on the Log-Sum NLMS algorithm, the Hybrid NLMS-NLMF algorithm further improves performance under low SNR conditions. It adeptly integrates the rapid convergence of NLMS with the benefits of NLMF in low SNR scenarios. Its cost function integrates statistical error metrics with sparse penalty terms, employing a mixed error function encompassing both mean square and fourth-order errors, along with a log-sum function as a sparsity constraint. This design enables the Hybrid NLMS-NLMF algorithm to excel across varying SNR conditions, achieving lower estimation errors and faster convergence rates.
- A comprehensive series of simulation experiments has been conducted to validate the effectiveness and advantages of the Log-Sum NLMS and Hybrid NLMS-NLMF algorithms. We also analyzed the computational complexity of each algorithm and theoretically proved that the proposed algorithms have low computational complexity. Our research offers novel perspectives and practical solutions for channel estimation in mmWave massive MIMO systems, opening new avenues for applying adaptive filtering techniques in RIS-assisted wireless communication frameworks.

The remainder of the paper is structured as follows: Section 2 introduces the channel model, while Section 3 outlines the adaptive filtering framework and elaborates on the proposed Log-Sum NLMS and Hybrid NLMS-NLMF algorithms, along with an analysis of their computational complexity. Section 4 evaluates the effectiveness of the proposed algorithms through simulation experiments. Finally, the conclusion is presented in Section 5.

## 2. SystemModel and Problem Formulation

### 2.1. System Model

**Figure 1:**
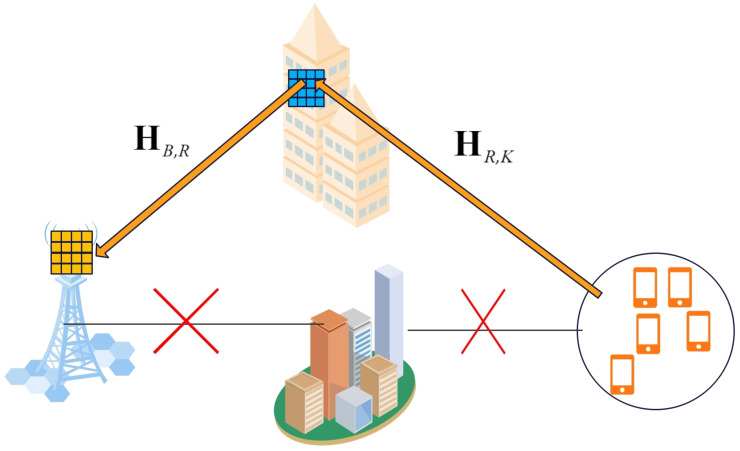
The RIS-aided wireless communication systems.

This research focuses on an uplink mmWave MIMO system facilitated by a reconfigurable intelligent surface (RIS), where the base station (BS) employs an antenna array containing *M* elements, and the RIS is equipped with a uniform planar array (UPA) composed of *N* elements. These arrays jointly serve *K* single-antenna users. Notably, the direct transmission path between the BS and users is assumed to be blocked. To overcome this challenge, the RIS, which is positioned on a tall structure [16], is used to facilitate communication between the BS and the users, as shown in Figure 1. In mmWave systems, where direct transmission paths are often unavailable due to severe path loss and blockage, the RIS is indispensable for establishing virtual line-of-sight (LoS) links. This capability ensures reliable communication and enhanced coverage in environments with limited direct connectivity.

The channel between the BS and the RIS is denoted as $H_{B,R}\in C^{M\times N}$, while the channel from the RIS to the *k*-th user is represented by $h_{R,K}\in C^{N\times 1}$. According to the widely adopted Saleh–Valenzuela channel model, $H_{B,R}$ is expressed as [17]

(1) $$H_{B,R}=\sqrt{\frac{MN}{L_{f}}}\sum_{l_{1}=1}^{L_{f}}\alpha_{l_{1}}b\left(\vartheta_{l_{1}}^{r},\psi_{l_{1}}^{r}\right)a{\left(\vartheta_{l_{1}}^{t},\psi_{l_{1}}^{t}\right)}^{T}.$$

Here, $L_{f}$ represents the number of propagation paths between the BS and the RIS. In this equation, $\alpha_{l_{1}}$ corresponds to the complex path gain, and $\vartheta_{l_{1}}^{r}\left(\psi_{l_{1}}^{r}\right)$ and $\vartheta_{l_{1}}^{t}\left(\psi_{l_{1}}^{t}\right)$ are the azimuth (or elevation) angles at the BS and RIS for the $l_{1}$-th path. Similarly, the channel from the RIS to the *k*-th user, $h_{R,K}$, can be written as

(2) $$h_{R,K}=\sqrt{\frac{N}{L_{g}}}\sum_{l_{2}=1}^{L_{g}}\alpha_{l_{2}}a\left(\vartheta_{l_{2}}^{r,k},\psi_{l_{2}}^{r,k}\right).$$

Here, $L_{g}$ denotes the number of propagation paths between the *k*-th user and the RIS. The parameters $\alpha_{l_{2}}$ and $\vartheta_{l_{2}}^{r,k}\left(\psi_{l_{2}}^{r,k}\right)$ represent the path gain and the azimuth (or elevation) angles at the RIS for the $l_{2}$-th path.

The normalized array steering vectors for the BS and RIS are denoted by $b\left(\vartheta ,\psi \right)\in C^{M\times 1}$ and $a\left(\vartheta ,\psi \right)\in C^{N\times 1}$, respectively.

For a typical UPA configuration with $N=N_{1}\times N_{2}$ (where $N_{1}$ and $N_{2}$ denote the number of elements in the vertical and horizontal directions), the steering vector a(ϑ,ψ) is formulated as [18]

(3) $$\begin{array}{cc} a(\vartheta ,\psi ) & =\frac{1}{\sqrt{N}}\left[e^{-j2\pi d\sin\left(\vartheta \right)\cos\left(\psi \right)n_{1}/\lambda }\right]\\ \; & ⊗\left[e^{-j2\pi d\sin\left(\psi \right)n_{2}/\lambda }\right]. \end{array}$$

Here, $n_{1}=\left[0,1,\ldots ,N_{1}-1\right]$ and $n_{2}=\left[0,1,\ldots ,N_{2}-1\right]$, where λ represents the carrier wavelength and *d* denotes the distance between antenna elements, typically set to d=λ/2. The cascaded channel for the *k*-th user is described by $h_{k}=H_{B,R}\mathrm{diag}\left(h_{R,K}\right)\in C^{M\times N}$.

Due to the high-frequency characteristics of mmWave channels, which are susceptible to severe path loss and blockage, the scattering environment surrounding the BS and RIS is constrained. This limitation results in only a few propagation paths being available between the transmitter and receiver, thereby causing angular-domain sparsity [19]. Through the use of virtual angular domain representation, the cascaded channel $h_{k}$ can be decomposed as follows [20]:

(4) $$h_{k}=U_{M}{\tilde{h}}_{k}U_{N}^{T}.$$

where ${\tilde{h}}_{k}$ represents the M×N angular cascaded channel. The matrices $U_{M}\in C^{M\times M}$ and $U_{N}\in C^{N\times N}$ are unitary dictionary matrices corresponding to the BS and the RIS. The sparsity of ${\tilde{h}}_{k}$ is defined by the total number of paths between the BS and RIS and between the users and the RIS. As a result, ${\tilde{h}}_{k}$ exhibits sparsity, containing only a few non-zero elements.

### 2.2. Problem Formulation

This part primarily addresses the formulation of the cascaded channel estimation problem. For channel estimation, each user transmits a predefined pilot signal, carefully designed to maintain orthogonality with the pilot signals of other users [21]. This approach enables the base station (BS) to distinguish between the channels of individual users effectively. The pilot signals are transmitted via the cascaded user–RIS-BS channel over *Q* time slots.The signal received at the BS for the *k*-th user during the *q*-th time slot (q=1,2,…,Q) can be expressed as

(5) $$\begin{array}{cc} y_{k,q} & =H_{B,R}\mathrm{diag}\left(\theta_{q}\right)h_{R,K}s_{k,q}+v_{k,q}\\ \; & =H_{B,R}\mathrm{diag}\left(h_{R,K}\right)\theta_{q}s_{k,q}+v_{k,q}. \end{array}$$

Here, $s_{k,q}$ denotes the pilot symbol transmitted by the *k*-th user, while $\theta q={\left[\theta_{q,1},\ldots ,\theta_{q,N}\right]}^{T}\in C^{N\times 1}$ corresponds to the reflection vector of the RIS during time slot *q*. The element $\theta_{q,n}$ indicates the reflection coefficient of the *n*-th RIS element (n=1,…,N). The noise term $v_{k,q}∼\mathrm{CN}\left(0,\sigma^{2}I_{M}\right)\in C^{M\times 1}$ represents complex Gaussian noise, with $\sigma^{2}$ denoting the noise variance. Taking into account the cascaded channel $h_{k}=H_{B,R}\mathrm{diag}\left(h_{R,K}\right)$, Equation (5) can be reformulated as

(6) $$y_{k,q}=h_{k}\theta_{q}s_{k,q}+v_{k,q}.$$

By stacking the received signals over all *Q* time slots and assuming $s_{k,q}=1$ [22], the overall measurement matrix $Y_{k}\in C^{M\times Q}$ can be written as

(7) $$Y_{k}=h_{k}\Theta +V_{k}.$$

Substituting Equation (4) into (7) gives

(8) $$Y_{k}=U_{M}{\tilde{h}}_{k}U_{N}^{T}\Theta +V_{k}.$$

Define ${\tilde{Y}}_{k}={\left(U_{M}^{H}Y_{k}\right)}^{H}\in C^{Q\times M}$ as the effective measurement matrix, ${\tilde{V}}_{k}={\left(U_{M}^{H}V_{k}\right)}^{H}\in C^{Q\times M}$ as the effective noise matrix, and $\tilde{\Theta }={\left(U_{N}^{T}\Theta \right)}^{H}\in C^{Q\times N}$ as the sensing matrix. The channel estimation model, based on compressed sensing (CS), is thus formulated as follows:

(9) $${\tilde{Y}}_{k}=\tilde{\Theta }{\tilde{h}}_{k}^{H}+{\tilde{V}}_{k}.$$

Using Equation (9), the angular cascaded channel for each user *k* can be estimated through standard compressed sensing techniques [23].

## 3. Proposed Algorithm

### 3.1. Adaptive Filter Framework to Solve CS Problem

**Figure 2:**
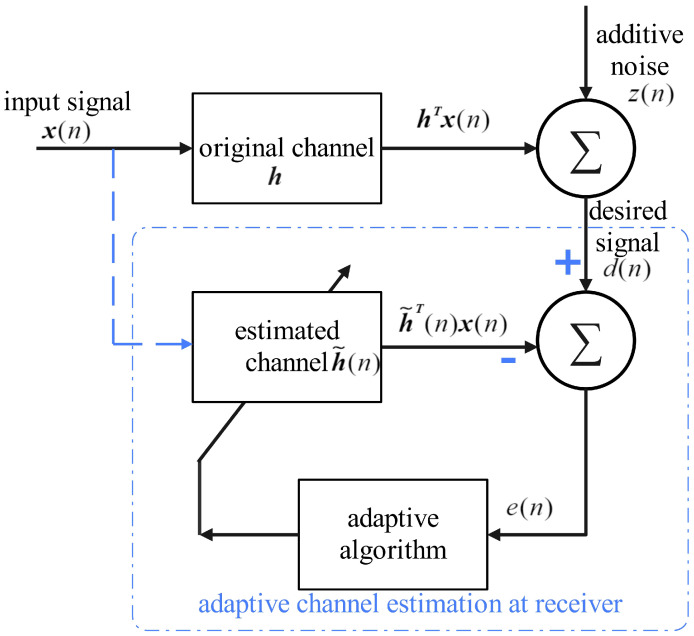
Adaptive filter framework.

Adaptive filtering techniques have attracted significant attention in both theoretical research and practical applications due to their exceptional effectiveness, ease of implementation, and reliable robustness [24]. As illustrated in Figure 2, a standard framework for an adaptive filter is shown, wherein the recursion error, which constitutes the cost function for each iteration, is defined as follows:

(10) $$e\left(n\right)=d\left(n\right)-{\tilde{h}}^{T}\left(n\right)x\left(n\right).$$

In this equation, $d\left(n\right)=h^{T}x\left(n\right)+z\left(n\right)$ denotes the desired output, whereas x(n) represents the input vector. z(n) represents the noise component, and $\tilde{h}\left(n\right)$ signifies the channel estimate at the *n*-th iteration, where *N* denotes the channel length. By seeking to minimize the recursion error presented in Equation (10), adaptive algorithms iteratively approximate the actual channel h [25].

Equation (9) from the previous section can be viewed as a classic example of sparse signal reconstruction, which can be addressed using traditional compressed sensing (CS) methods [26]. If the CS problem is treated as an adaptive estimation task, the relationships between the variables are outlined in Table 1. Therefore, Equation (9) can be effectively approached within the adaptive filtering framework.

### 3.2. Proposed Hybrid NLMS-NLMF Algorithm

In Section 2.2, we obtained the following expression: ${\tilde{Y}}_{k}=\tilde{\Theta }{\tilde{h}}_{k}^{H}+{\tilde{V}}_{k}$. Let $\hat{h}\left(n,k\right)$ denote the channel estimation at the *n*-th iteration. The Mean Squared Observation Error (MSOE) cost function for this estimation is given by

(11) $$\begin{array}{c} J\left(n,k\right)=E\left\{{∥{\tilde{Y}}_{k}-\tilde{\Theta }{\hat{h}}^{H}\left(n,k\right)∥}_{2}^{2}\right\}. \end{array}$$

To minimize J(n,k), we compute its gradient with respect to $\hat{h}\left(n,k\right)$:

(12) $$\begin{array}{c} \frac{\partial J(n,k)}{\partial \hat{h}\left(n,k\right)}=-2{\tilde{\Theta }}^{H}{\tilde{Y}}_{k}+2{\tilde{\Theta }}^{H}\tilde{\Theta }\hat{h}\left(n,k\right). \end{array}$$

Using the standard normalized least mean squares (NLMS) approach, we derive an update rule by taking a gradient descent step:

(13) $$\begin{array}{c} \hat{h}\left(n+1,k\right)=\hat{h}\left(n,k\right)+\mu_{0}\frac{{\tilde{\Theta }}^{H}e\left(n,k\right)}{∥\tilde{\Theta }{∥}^{2}+\varepsilon_{0}}, \end{array}$$

where $\varepsilon_{0}$ is a small constant to ensure stability.

To improve sparsity in the estimation process, we integrate a log-sum penalty term into the cost function of the NLMS algorithm. Theoretical analyses demonstrate that the log-sum penalty is more closely aligned with the $\ell_{0}$-norm than the $\ell_{1}$-norm. This property allows it to efficiently penalize the number of nonzero coefficients, thereby promoting a faster convergence of the channel coefficients toward zero. Consequently, the application of this penalty accelerates the convergence of the algorithm. The modified cost function is expressed as follows:

(14) $$\begin{array}{c} J\left(n,k\right)=E\left\{{∥{\tilde{Y}}_{k}-\tilde{\Theta }{\hat{h}}^{H}\left(n,k\right)∥}^{2}\right\}+\gamma_{0}\sum_{i=1}^{N}\log(\alpha +\beta |\hat{h}\left(n,k\right)\left|\right), \end{array}$$

where $\gamma_{0}$ is the weight of the sparsity constraint, and α and β are small constants to prevent singularities.

The gradient of this modified cost function J(n,k) with respect to $\hat{h}\left(n,k\right)$ is

(15) $$\begin{array}{c} \frac{\partial J(n,k)}{\partial \hat{h}\left(n,k\right)}=-2{\tilde{\Theta }}^{H}{\tilde{Y}}_{k}+2{\tilde{\Theta }}^{H}\tilde{\Theta }\hat{h}\left(n,k\right)-\gamma_{0}\frac{\mathrm{sgn}(\hat{h}\left(n,k\right))}{\varepsilon_{1}+\left|\hat{h}\left(n,k\right)\right|}, \end{array}$$

where $\varepsilon_{1}$ is another small constant to prevent division by zero in the log-sum penalty term.

The Log-Sum NLMS update rule is then given by

(16) $$\begin{array}{c} \hat{h}\left(n+1,k\right)=\hat{h}\left(n,k\right)+\mu_{0}\frac{{\tilde{\Theta }}^{H}e\left(n,k\right)}{∥\tilde{\Theta }{∥}^{2}+\varepsilon_{0}}-\rho_{0}\frac{\mathrm{sgn}(\hat{h}\left(n,k\right))}{\varepsilon_{1}+\left|\hat{h}\left(n,k\right)\right|}, \end{array}$$

where $\mu_{0}$ is the step size for channel estimation, and $\rho_{0}$ is the parameter controlling the sparsity-inducing penalty.

This equation includes both the adaptive channel estimation term and the sparsity-inducing log-sum penalty, ensuring better estimation performance in sparse mmWave channels.

The Log-Sum NLMS algorithm, based on second-order statistical errors, demonstrates considerable sensitivity to noise interference, resulting in suboptimal estimation performance, especially in low SNR environments. To address this issue, this paper presents the Hybrid NLMS-NLMF algorithm, which integrates the advantages of both the NLMS and NLMF algorithms. This integration allows the adaptive filtering process to attain a low steady-state estimation error and rapid convergence, even under low SNR conditions. The Hybrid NLMS-NLMF algorithm substantially enhances channel estimation accuracy across various SNR regimes; however, this enhancement comes with increased computational complexity. The algorithm formulates a cost function that combines a statistical error term with a sparse penalty term. The statistical error term includes mean square and fourth-order mean errors, whereas the sparse penalty term utilizes a log-sum function. The key difference here is that the error term e(n,k) undergoes a nonlinear filtering process, denoted as f(e(n,k)), where f(·) is a nonlinear function used to mitigate outlier effects.

The update rule for Hybrid NLMS-NLMF is then

(17) $$\begin{array}{c} \hat{h}\left(n+1,k\right)=\hat{h}\left(n,k\right)+\mu {\tilde{\Theta }}^{H}f\left(e\left(n,k\right)\right)-\rho \frac{\mathrm{sgn}(\hat{h}\left(n,k\right))}{\varepsilon_{2}+\left|\hat{h}\left(n,k\right)\right|}. \end{array}$$

So, the update equation for RIS-assisted mmWave massive MIMO channel estimation based on Hybrid NLMS-NLMF can be obtained as

(18) $$\begin{array}{cc} \hat{h}\left(n+1,k\right)= & \hat{h}\left(n,k\right)+\mu_{1}\frac{\alpha {\tilde{\Theta }}^{H}e\left(n,k\right)}{∥\tilde{\Theta }{∥}^{2}+\varepsilon_{3}}\\ \; & +\mu_{2}\frac{\left(1-\alpha \right){\tilde{\Theta }}^{H}e^{3}\left(n,k\right)}{∥\tilde{\Theta }{∥}^{2}+\varepsilon_{3}}-\rho \frac{\mathrm{sgn}(\hat{h}\left(n,k\right))}{\varepsilon_{2}+\left|\hat{h}\left(n,k\right)\right|}. \end{array}$$

In this context, $\mu_{1}$ denotes the step size related to the NLMS, whereas $\mu_{2}$ pertains to the step size employed in the NLMF. The parameter ρ is the zero attractor, and $\varepsilon_{2}$ functions as a reweighting factor. The Hybrid NLMS-NLMF algorithm improves the convergence speed and estimation precision equilibrium by utilizing non-uniform step sizes ($\mu_{1}$ and $\mu_{2}$). By optimally adjusting the parameters α and the corresponding step sizes, this algorithm can attain enhanced channel estimation accuracy across a wide range of signal-to-noise ratio (SNR) scenarios.

The zero attractor parameter, denoted by ρ, is critical for determining convergence behavior. It regulates the speed at which the weight coefficients converge to zero. With increasing channel sparsity, a higher value of the zero attractor becomes necessary. Increased values of ρ impose stricter sparsity constraints, pulling the weight coefficients toward zero more rapidly. Conversely, a lower zero attractor is beneficial when the channel is less sparse, resulting in weaker sparsity constraints. This work specifically focuses on mmWave channels, which are widely recognized for their inherent sparsity due to limited scattering and dominant propagation paths. The parameter ρ is designed to be adjustable, enabling the algorithm to adapt flexibly to variations in channel sparsity levels.

The RIS-assisted mmWave massive MIMO channel estimation method based on the Hybrid NLMS-NLMF algorithm can be summarized in Algorithm 1.
**Algorithm 1** Hybrid NLMS-NLMF-based cascaded channel estimation.   **Input:**  Measurement matrix: ${\tilde{Y}}_{k}$  Sensing matrix: $\tilde{\Theta }$  **Ouput:** Estimated angular cascaded channel: $\hat{h}\left(n,k\right)$   **Initialization:** $\hat{h}\left(n,k\right)=0$   **for** k=1,2,⋯,K **do**     **for** n=0,1,⋯ max-iteration **do**      $e\left(n,k\right)={\tilde{Y}}_{k}-\tilde{\Theta }\hat{h}\left(n,k\right)$      $\begin{array}{c} \hat{h}\left(n+1,k\right)=\hat{h}\left(n,k\right)+\mu_{1}\frac{\alpha {\tilde{\Theta }}^{H}e\left(n,k\right)}{∥\tilde{\Theta }{∥}^{2}+\varepsilon_{3}}\\ +\mu_{2}\frac{\left(1-\alpha \right){\tilde{\Theta }}^{H}e^{3}\left(n,k\right)}{∥\tilde{\Theta }{∥}^{2}+\varepsilon_{3}}-\rho \frac{\mathrm{sgn}(\hat{h}\left(n,k\right))}{\varepsilon_{2}+\left|\hat{h}\left(n,k\right)\right|} \end{array}$     **end for**  **end for**  **return** $\hat{h}\left(n,k\right)$

### 3.3. Computational Complexity Analysis

This research examines the computational complexities associated with four adaptive filtering algorithms: Log-Sum NLMS, Hybrid NLMS-NLMF, SHAFA [27], and SEFWLMS [28]. The computational complexity of these algorithms are presented in Table 2. By assessing the complexities of these algorithms in terms of multiplication and addition operations, we can distinctly identify their performance characteristics. The Log-Sum NLMS algorithm demonstrates a multiplication complexity of 5N+1 and an addition complexity of 5N, making it the most computationally efficient among the algorithms considered. The Hybrid NLMS-NLMF algorithm proposed in this study improves upon its predecessor with a multiplication complexity of 6N+6 and an addition complexity of 6N. Although its computational complexity slightly exceeds that of the Log-Sum NLMS algorithm, it substantially improves estimation accuracy and robustness by integrating a nonlinear least mean square filtering mechanism. The Hybrid NLMS-NLMF algorithm exhibits superior performance across the entire SNR range, leveraging its noise-robust design for low SNR conditions and achieving consistent improvements in high SNR scenarios, as confirmed by the simulation results. The SHAFA algorithm possesses a multiplication complexity of 6N+8 and an addition complexity of 6N. This algorithm strikes a balance between complexity and performance. Among the four algorithms, the SEFWLMS algorithm exhibits the highest computational complexity, characterized by a multiplication complexity of 2WN+4N+W and an addition complexity of WN+2N, where *W* denotes the window size.

## 4. Simulations

The primary purpose of the simulations is to evaluate the performances of the proposed Log-Sum NLMS and Hybrid NLMS-NLMF algorithms in estimating sparse channels in RIS-assisted mmWave massive MIMO systems. Specifically, the experiments aim to validate the algorithms’ performances in terms of NMSE, compare their convergence speed with previously reported algorithms such as NLMS, SHAFA [27], and SEFWLMS [28], and analyze the influence of algorithm parameters on estimation accuracy. The simulation environment is based on the widely used Saleh–Valenzuela channel model, which captures the sparse characteristics of mmWave channels. Channel sparsity is controlled by adjusting the number of propagation paths, while both the BS and RIS are configured with uniform planar arrays (UPAs). These configurations provide a realistic environment for evaluating the proposed algorithms. For the NLMS algorithms, the parameter is configured as $\mu_{\mathrm{NLMS}}=5\times 10^{-2}$. The parameters for the SEFWLMS algorithm are assigned as $\mu_{\mathrm{SEFWLMS}}=7\times 10^{-1}$ and $\rho_{\mathrm{SEFWLMS}}=1\times 10^{-10}$, and the SHAFA algorithm employs $\rho_{\mathrm{SHAFA}}=9\times 10^{-10}$. The Log-Sum NLMS algorithm utilizes parameters $\mu_{0}=9\times 10^{-2}$ and $\rho_{0}=1\times 10^{-10}$. In the case of the Hybrid NLMS-NLMF algorithm, the parameters are set as follows: $\alpha =3\times 10^{-1}$, $\mu_{1}=6\times 10^{-1}$, $\mu_{2}=4\times 10^{-1}$, and $\rho =2\times 10^{-10}$. These settings ensure optimal performance for each algorithm under the same simulation conditions.

Throughout all the simulation experiments, the normalized mean square error (NMSE) served as the principal metric for assessing the channel estimation performance in RIS-assisted massive MIMO systems, defined as follows:

(19) $$\mathrm{NMSE}=\frac{{∥{\tilde{h}}_{k}-\hat{h}\left(n,k\right)∥}_{2}^{2}}{{∥{\tilde{h}}_{k}∥}_{2}^{2}},$$

where ${\tilde{h}}_{k}$ represents the true angular cascaded channel between the *k*-th user and the base station, while $\hat{h}\left(n,k\right)$ denotes the estimated channel value following the *n*-th iteration of the algorithm. The notation ${∥\cdot ∥}_{2}$ refers to the $\ell_{2}$ norm operation.

Additionally, the simulation parameters used in the experiments are summarized in Table 3. This table provides a comprehensive overview of the experimental setup, including the number of antennas, RIS elements, users, time slots for channel estimation, path loss exponents, propagation paths, and antenna spacing.

**Figure 3:**
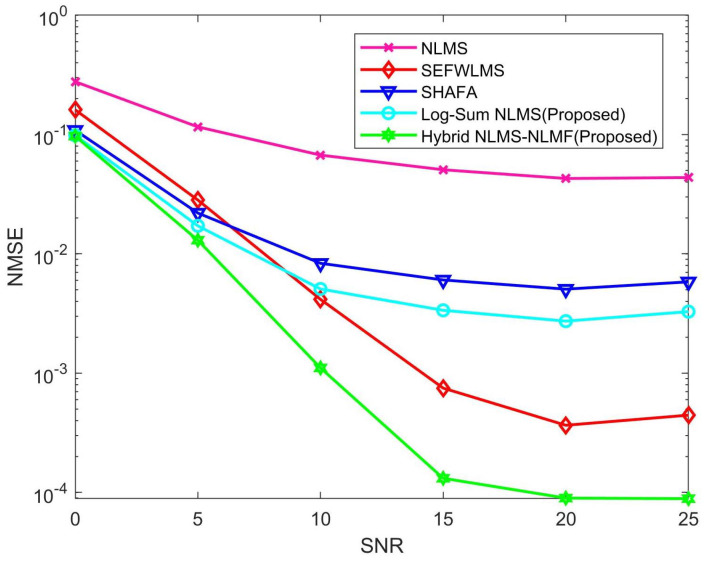
NMSE of different estimation algorithms versus SNR.

Figure 3 illustrates the performance of various channel estimation algorithms in terms of normalized mean square error (NMSE) across multiple SNR scenarios over 400 iterations. The traditional NLMS algorithm demonstrates the poorest channel estimation performance, primarily due to its inability to leverage the sparse characteristics of the channel, resulting in less accurate estimates, particularly with limited iterations and pilots. In contrast, other algorithms incorporate sparsity constraints that significantly enhance estimation accuracy. The Hybrid NLMS-NLMF algorithm stands out, exhibiting exceptional performance even under low SNR conditions, and its advantage over other algorithms increases as SNR rises, confirming its superiority across the entire range. While the SEFWLMS algorithm also performs well at high SNR, its improvements are less pronounced compared to the Hybrid NLMS-NLMF. At a constant SNR of 20 dB, the NMSE values for NLMS, SEFWLMS, SHAFA, Log-Sum NLMS, and Hybrid NLMS-NLMF are approximately $5\times 10^{-2}$, $2.1\times 10^{-4}$, $5\times 10^{-3}$, $2.6\times 10^{-3}$, and $8.5\times 10^{-5}$, respectively.

**Figure 4:**
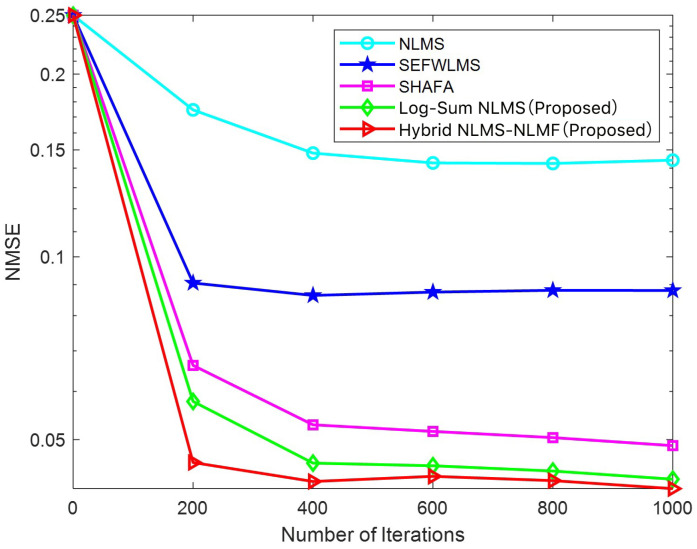
NMSE of different estimation algorithms versus number of iterations at an SNR of 3 dB.

**Figure 5:**
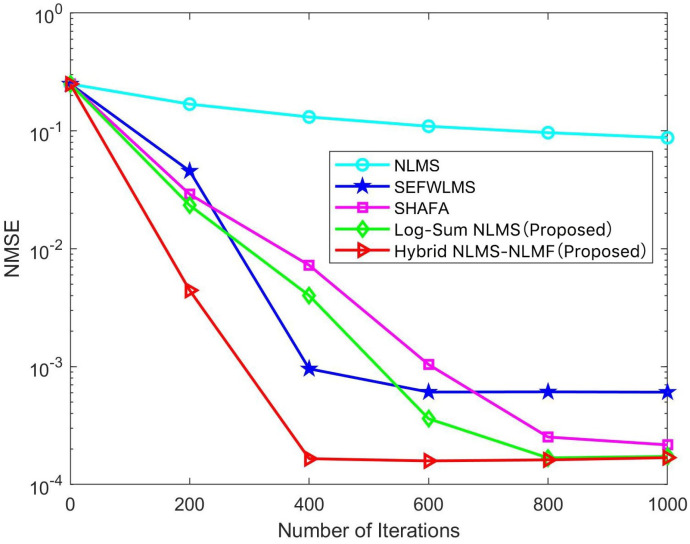
NMSE of different estimation algorithms versus number of iterations at an SNR of 13 dB.

Figure 4 and Figure 5 illustrate the variation in NMSE for different adaptive filtering estimation algorithms as the iteration count increases, specifically at SNR levels of 3 dB and 13 dB. At 3 dB, the Hybrid NLMS-NLMF algorithm exhibits faster convergence and lower estimation error than other methods. This trend continues as the SNR increases to 13 dB, indicating that the proposed algorithm is robust across varying SNR conditions. The Hybrid NLMS-NLMF algorithm combines the rapid convergence of NLMS with the steady-state performance of NLMF, enhancing sparse constraints through a log-sum penalty. This additional term accelerates the convergence of channel values close to zero, achieving an optimal balance between speed and estimation accuracy. Overall, the Hybrid NLMS-NLMF consistently outperforms the Log-Sum NLMS, SHAFA, SEFWLMS, and NLMS algorithms. It is important to note that the parameters of the proposed algorithm significantly influence estimation performance. Therefore, a detailed analysis of α, ρ, $\mu_{1}$, and $\mu_{2}$ will be conducted in the subsequent sections.

**Figure 6:**
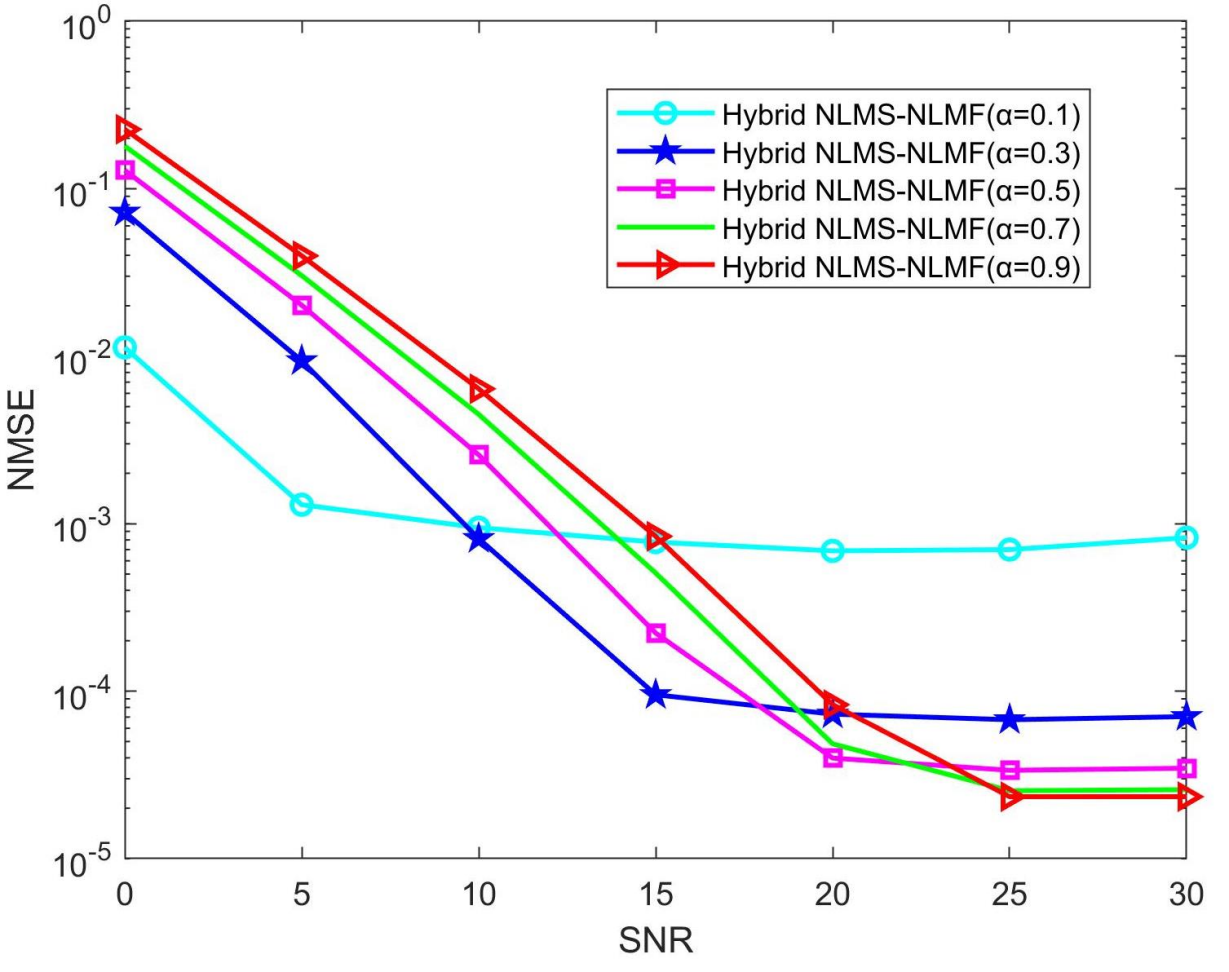
Impact of the parameter α on the NMSE performance of the Hybrid NLMS-NLMF algorithm.

Figure 6 illustrates the performance variation of the Hybrid NLMS-NLMF algorithm as the parameter α ranges from 0.1 to 0.9. Notably, decreasing α improves accuracy in low SNR conditions, though this benefit diminishes at higher SNR levels, where accuracy tends to decline. This finding supports previous analyses, indicating that lower α values increase sensitivity to mean fourth error, thereby reducing estimation errors in low SNR scenarios. Conversely, higher α values enhance the influence of mean square error, improving accuracy in high SNR environments. As SNR increases, a larger α is recommended to strengthen the NLMS component of the Hybrid NLMS-NLMF algorithm, thereby enhancing channel estimation performance. Therefore, identifying an optimal α based on current SNR conditions is crucial for achieving peak performance in practical applications.

**Figure 7:**
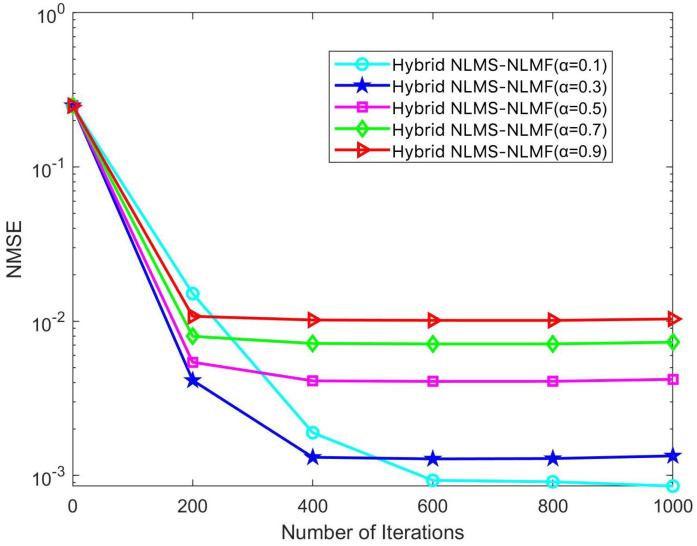
Impact of the parameter α on the convergence trend of the Hybrid NLMS-NLMF algorithm at an SNR of 10 dB.

Figure 7 illustrates how varying α values influence the convergence properties of the Hybrid NLMS-NLMF algorithm at an SNR of 10 dB. For all tested α values, the NMSE shows an initial rapid decline, indicating the algorithm’s quick adaptability during the early stages of channel estimation. After several iterations, the NMSE stabilizes, signifying that the algorithm has reached a steady state. Larger α values result in faster convergence but higher estimation errors, while smaller values reduce error at the cost of a slower convergence rate. Notably, when α is set to 0.1, the algorithm demonstrates the slowest convergence rate but achieves minimal steady-state estimation error. Thus, α is crucial for balancing convergence speed and estimation accuracy, highlighting the importance of its selection to optimize the Hybrid NLMS-NLMF algorithm’s performance for specific applications.

**Figure 8:**
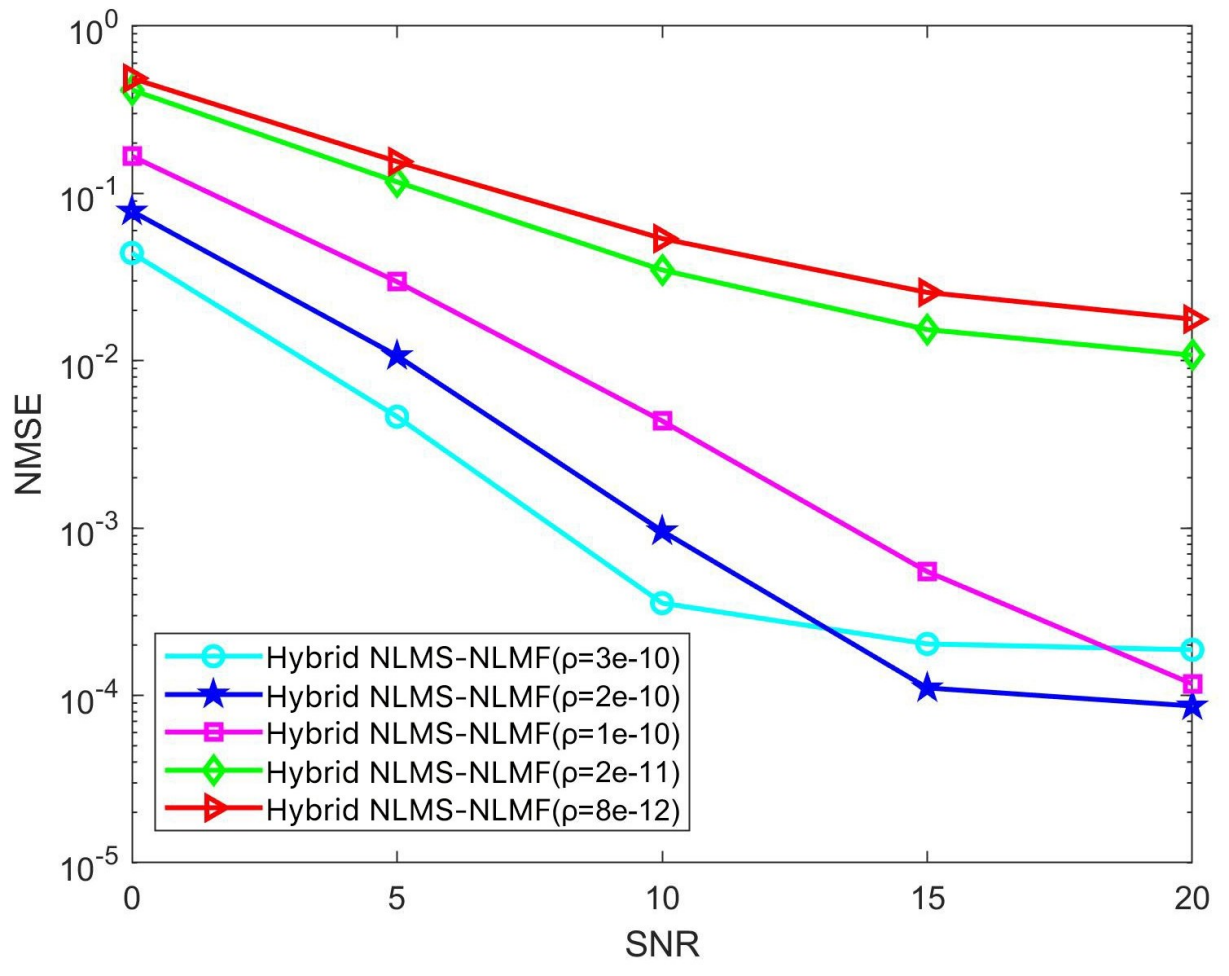
Impact of the parameter ρ on the NMSE performance of the Hybrid NLMS-NLMF algorithm.

**Figure 9:**
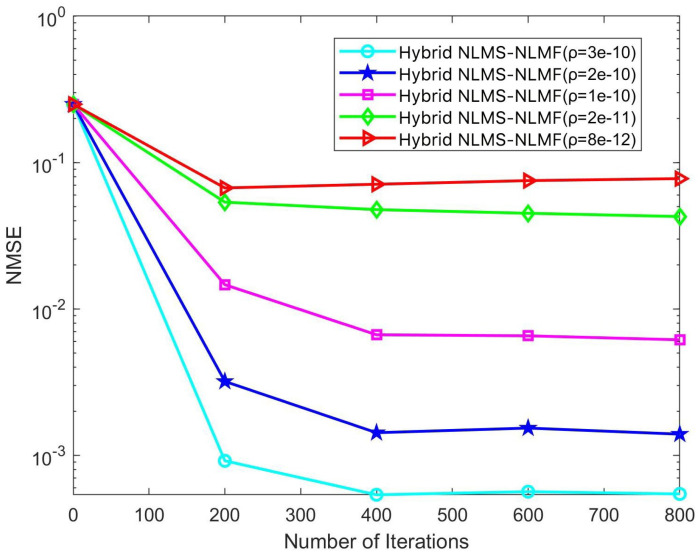
Impact of the parameter ρ on the convergence trend of the Hybrid NLMS-NLMF algorithm at an SNR of 10 dB.

In the iterative implementation of the Hybrid NLMS-NLMF algorithm, a zero attraction term governed by the parameter ρ is included. This study investigates the impact of ρ on convergence efficiency and steady-state error by varying it across five specific values: $3\times 10^{-10}$, $2\times 10^{-10}$, $1\times 10^{-10}$, $2\times 10^{-11}$, and $8\times 10^{-12}$. The effects of these adjustments are illustrated in Figure 8 and Figure 9. Figure 8 highlights how different ρ values influence NMSE performance across various SNR conditions. Notably, NMSE performance improves with increasing ρ, especially in low SNR scenarios. This enhancement is due to the stricter sparsity constraints imposed by higher ρ values, which facilitate more rapid convergence of the weight coefficients toward zero. Optimizing ρ can thus significantly enhance the algorithm’s performance across a broad range of SNR conditions, improving its effectiveness in estimating complex and dynamic communication channels.

Figure 9 illustrates how different ρ values affect the convergence dynamics of the Hybrid NLMS-NLMF algorithm at an SNR of 10 dB. The convergence trajectories demonstrate that the zero attractor ρ directly influences the speed at which weight coefficients converge to zero. Specifically, increasing ρ from $8\times 10^{-12}$ to $3\times 10^{-10}$ significantly accelerates convergence toward a stable state. Optimal performance occurs at $\rho =3\times 10^{-10}$, where the lowest steady-state error and fastest convergence rate are achieved. Analysis of these figures indicates that ρ is a critical parameter in the performance of the Hybrid NLMS-NLMF method. Its selection significantly impacts the ability to impose sparsity constraints, affecting both convergence speed and the accuracy of steady-state estimates. Therefore, when designing the Hybrid NLMS-NLMF algorithm, careful selection of ρ is essential, considering channel sparsity and the required level of estimation precision.

**Figure 10:**
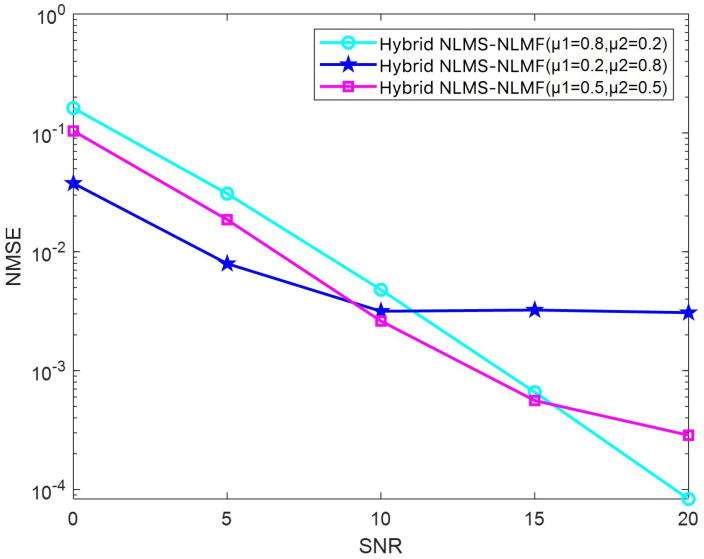
Impact of the parameter μ on the NMSE performance of the Hybrid NLMS-NLMF algorithm.

Figure 10 illustrates the NMSE curve for the Hybrid NLMS-NLMF algorithm as it varies with the SNR across three distinct step size configurations ($\mu_{1}$ and $\mu_{2}$). When $\mu_{1}=0.8$ and $\mu_{2}=0.2$, the larger step size in the NLMS term allows the algorithm to leverage second-order errors more effectively for channel estimation. This configuration yields positive results in high SNR environments, facilitating rapid convergence. However, the larger step size in low SNR scenarios leads to suboptimal performance. In contrast, with $\mu_{1}=0.2$ and $\mu_{2}=0.8$, the NLMS term adopts a smaller step size, while the NLMF term uses a larger one, enhancing performance in low SNR conditions. The higher $\mu_{2}$ emphasizes the NLMF term during updates, improving estimation accuracy. However, as SNR increases, the NMSE reduction rate diminishes due to the decreasing advantage of NLMF over NLMS in high SNR conditions. When $\mu_{1}=\mu_{2}=0.5$, a balanced step size harmonizes the effects of second-order and fourth-order errors, demonstrating the effectiveness of integrating the NLMS and NLMF algorithms. This configuration shows commendable performance across varying SNR levels.

**Figure 11:**
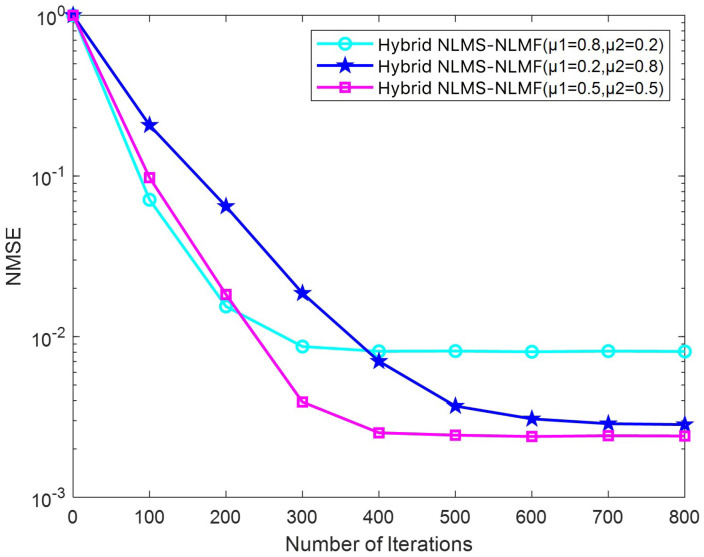
Impact of the parameter μ on the convergence trend of the Hybrid NLMS-NLMF algorithm at an SNR of 10 dB.

Figure 11 presents the changes in the NMSE relative to the number of iterations for the Hybrid NLMS-NLMF algorithm across different step size configurations. A higher $\mu_{1}$ leads to swift convergence, with a notable reduction in the NMSE during the initial iterations. This rapid adaptation is attributed to the increased weighting of the NLMS component, which, while promoting a quick adjustment, can compromise the final estimation accuracy. Conversely, the convergence rate declines as $\mu_{2}$ increases, but the algorithm achieves higher estimation accuracy. When $\mu_{1}$ and $\mu_{2}$ are equal, the convergence speed is intermediate, reflecting a balanced influence of both components. The selection of step sizes $\mu_{1}$ and $\mu_{2}$ is crucial. Although a larger $\mu_{1}$ accelerates initial convergence, excessively high values may impair final accuracy. In contrast, a larger $\mu_{2}$ improves final accuracy but may slow convergence. Proper adjustment of these parameters enables the Hybrid NLMS-NLMF algorithm to adapt effectively to various SNR conditions, achieving both rapid convergence and precise estimations.

**Figure 12:**
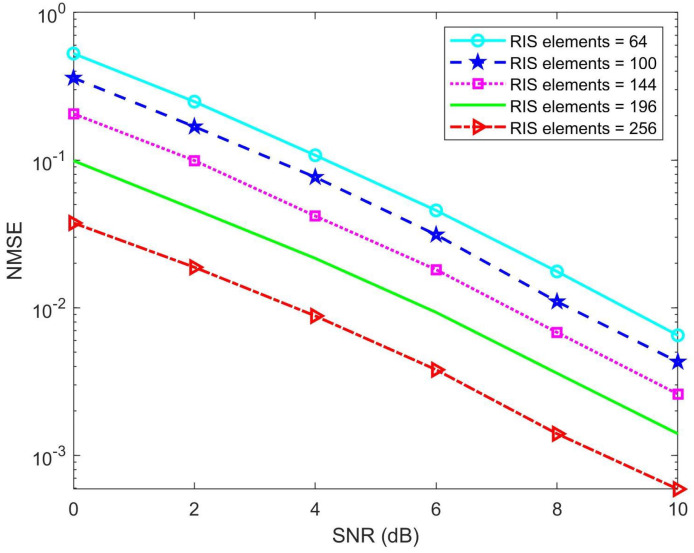
Impact of RIS elements on the NMSE performance of the Hybrid NLMS-NLMF algorithm.

To evaluate the influence of the RIS on the proposed algorithm, we conducted simulations by varying the number of RIS elements. Figure 12 shows the relationship between the normalized mean square error (NMSE) and the signal-to-noise ratio (SNR) for different numbers of RIS elements, specifically 64, 100, 144, 196, and 256. The results demonstrate that increasing the number of RIS elements significantly reduces the NMSE, indicating a substantial improvement in the performance of the proposed algorithm. This analysis highlights the effectiveness of increasing the number of RIS elements in enhancing channel estimation accuracy and improving the overall system performance in RIS-assisted millimeter-wave systems.

## 5. Conclusions

This study addresses the challenges of channel estimation in RIS-assisted mmWave massive MIMO systems. We present two innovative algorithms, Log-Sum NLMS and Hybrid NLMS-NLMF, designed to exploit the inherent sparsity of the channel. Notably, the computational complexity of the proposed algorithms is relatively low, making them suitable for practical applications. The Hybrid NLMS-NLMF algorithm exhibits remarkable performance in high SNR environments while maintaining robust estimation accuracy even under low SNR conditions. Simulation results demonstrate that these algorithms significantly outperform traditional NLMS and other adaptive sparse filtering methods, highlighting improved estimation accuracy and accelerated convergence rates. These findings offer critical insights for optimizing future mmWave communication systems and open new avenues for research in adaptive filtering applications within RIS-assisted wireless communication systems.

## Figures and Tables

**Table 1:** Correspondences between variables in adaptive filtering and compressed sensing problems.

Adaptive Filter	CS Problem
x(n)	$\tilde{\Theta }$
$\tilde{h}\left(n\right)$	${\tilde{h}}_{k}^{H}$
d(n)	${\tilde{Y}}_{k}$

**Table 2:** Computational complexity.

Algorithms	Multiplications	Additions
Log-Sum NLMS	5N+1	5N
Hybrid NLMS-NLMF	6N+6	6N
SHAFA	6N+8	6N
SEFWLMS	2WN+4N+W	WN+2N

**Table 3:** Channel parameter settings.

Parameter	Value
BS antennas	64
RIS elements	256
Number of users	16
Path loss exponent between the BS and the RIS	−2.2
Path loss exponent between the RIS and the users	−2.8
The number of time slots for channel estimation	256
The number of paths between the BS and the RIS	5
The number of paths between the RIS and the user	8
Antenna spacing	λ/2 (λ denotes wavelength)

## Data Availability

Data is contained within the article.
